# Using a 3D Silicon Micro-Channel Device and Raman Spectroscopy for the Analysis of Whole Blood and Abnormal Blood

**DOI:** 10.3390/mi15010021

**Published:** 2023-12-22

**Authors:** Chao-Ching Chiang, Song-Jeng Huang, Philip Nathaniel Immanuel, Jun-Han Lan, Fang-Yuh Lo, Kung-Chia Young

**Affiliations:** 1 Department of Mechanical Engineering, National Taiwan University of Science and Technology, Taipei 10607, Taiwan; shoo6667@hotmail.com (C.-C.C.); nathaniel.philip@gmail.com (P.N.I.); langer10203139@gmail.com (J.-H.L.); 2 Department of Physics, National Taiwan Normal University, Taipei 10611, Taiwan; fangyuhlo@ntnu.edu.tw; 3 Department of Medical Laboratory Science and Biotechnology, National Cheng Kung University, Tainan 70101, Taiwan

**Keywords:** blood testing, biological information, hemoglobin, Raman spectroscopy, 3D silicon micro-channel device

## Abstract

Blood testing is a crucial application in the field of clinical studies for disease diagnosis and screening, biomarker discovery, organ function assessment, and the personalization of medication. Therefore, it is of the utmost importance to collect precise data in a short time. In this study, we utilized Raman spectroscopy to analyze blood samples for the extraction of comprehensive biological information, including the primary components and compositions present in the blood. Short-wavelength (532 nm green light) Raman scattering spectroscopy was applied for the analysis of the blood samples, plasma, and serum for detection of the biological characteristics in each sample type. Our results indicated that the whole blood had a high hemoglobin content, which suggests that hemoglobin is a major component of blood. The characteristic Raman peaks of hemoglobin were observed at 690, 989, 1015, 1182, 1233, 1315, and 1562–1649 cm^−1^. Analysis of the plasma and serum samples indicated the presence of β-carotene, which exhibited characteristic peaks at 1013, 1172, and 1526 cm^−1^. This novel 3D silicon micro-channel device technology holds immense potential in the field of medical blood testing. It can serve as the basis for the detection of various diseases and biomarkers, providing real-time data to help medical professionals and patients better understand their health conditions. Changes in biological data collected in this manner could potentially be used for clinical diagnosis.

## 1. Introduction

Conventional blood tests are performed with optical microscopy. This process is time consuming and may lessen the accuracy of blood sample analysis. Raman spectroscopy is one of the emerging laser-based blood testing methods that relies upon nonlinear scattering. This method has advantages that make it adaptable for a wide range of blood tests because it requires a small volume of blood for testing and analysis if rapid and high-precision result interpretation is possible. These factors simplify the process of collecting and evaluating blood-related data. The effectiveness of Raman spectroscopy in clinical applications has been verified [1,2].

In Raman spectroscopy, the wavenumber difference between incident and scattered light is compared to determine the changes in their energy levels and the sample’s lattice vibration characteristics. These observations can be used to analyze the composition and structure of the sample. Therefore, the Raman peak values can indicate a sample’s molecular structure [3,4].

Raman spectroscopy can be applied in blood testing to determine the presence of certain diseases in individuals. There are distinct differences in the Raman characteristic peak positions and amplitudes between infected and uninfected blood samples. This makes Raman spectroscopy a valuable tool for rapid pathogen screening and identification. Reliable, rapid disease screening is necessary for the detection of emerging viruses, such as COVID-19, which will eventually overlap with the influenza virus infection [5]. Moreover, the acquisition of blood information through Raman spectroscopy has enabled the rapid and precise diagnosis of clinical diseases, such as liver disease, diabetes, cancer, allergies, genetic disorders, anemia, and leukemia [6,7,8].

In the present study, we fabricated a 3D silicon micro-channel device (3D SMCD) into which we introduced blood and calcium coagulants for mixing. The primary Raman peaks in whole blood were attributed to hemoglobin, while other Raman peaks were found that corresponded to amino acids and other components [9]. In contrast, the primary Raman peaks of plasma and serum corresponded to β-carotene [10]. A prothrombin time (PT) experiment was also conducted to compare the Raman spectroscopic results of blood samples with and without a calcium-containing coagulant. Coagulation or clotting, in which blood transforms from a liquid to a non-flowing gel, is a critical process in clinical hemostasis [11,12]. This phenomenon can be confirmed by using a transmission Raman spectrometer in the mixing zone of the 3D SMCD [13]. In advancing blood testing methodologies, this study emphasizes the unique contributions resulting from integrating 3D SMCD with Raman spectroscopy for blood sample analysis. By matching these two technologies, our approach goes beyond conventional methods [14], offering a novel and efficient means for comprehensive blood analysis. The 3D SMCD, meticulously designed for controlled blood and calcium coagulant mixing, introduces a level of precision and reliability that is crucial for accurate diagnostics. This incorporation of technologies not only streamlines the analytical process but also opens avenues for deeper insights into blood composition. Through this integrated platform, we aim to address the shortcomings of traditional blood testing methods and pave the way for a more effective, rapid, and precise diagnosis of various clinical conditions.

## 2. Sample Preparation for 3D SMCD

### 2.1. Fabrication of 3D SMCD

**Figure 1:**
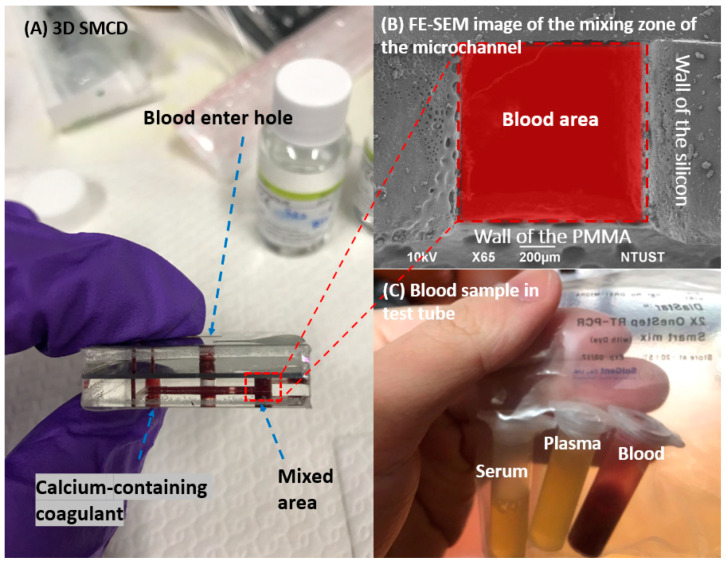
Preparation of blood samples using 3D SMCD (**A**) 3D SMCD; (**B**) FE-SEM image of the microchannel mixing zone; (**C**) blood sample in a test tube.

The 3D SMCD (sandwich structure—Poly(methyl methacrylate), PMMA/Silicon/PMMA) heterogeneous structure [13] was fabricated using a low-temperature bonding technique. The 3D SMCD structure comprises a blood inlet and an outlet connected through the middle layer of the microchannel to facilitate the entry of blood into the mixing zone. Both the upper and lower layers had their own mixing zone. The blood mixing area was bonded to the lower layer of the 3D SMCD (Figure 1A,B). This helps prevent product breakage or blood leakage. A dedicated circulation and mixing system was developed for the microchannel device by integrating a 3D SMCD with a rapid withdrawal device. A micro-thin film pump was employed to drive the liquids, including the blood and coagulation factors, enabling the automatic transport of these substances for testing. This design ensured thorough mixing of any fluids within the microchannel device’s mixing zone, minimizing the potential for errors in blood test data interpretation due to human-operational factors.

The specialized pump offers the flexibility of quantitative or variable settings within a single timeframe. Its compact, ultra-thin design with a high flow rate makes it suitable for integration into devices with a limited volume. The experimental procedure began with the initiation of automatic circulation and placing the microchannel device in the rapid-release device. Blood and coagulation factors were then injected into fluorinated ethylene propylene (ETFE) resin tubes with diameters of 2.0 mm and 4.0 mm for leakage testing. Subsequently, the micropump was activated to drive the liquids from the tubes into the microchannel device at a maximum flow rate of 15 mL/min, using water at 25 °C. The micropump was operated at a default rate of 50 Hz.

**Figure 2:**
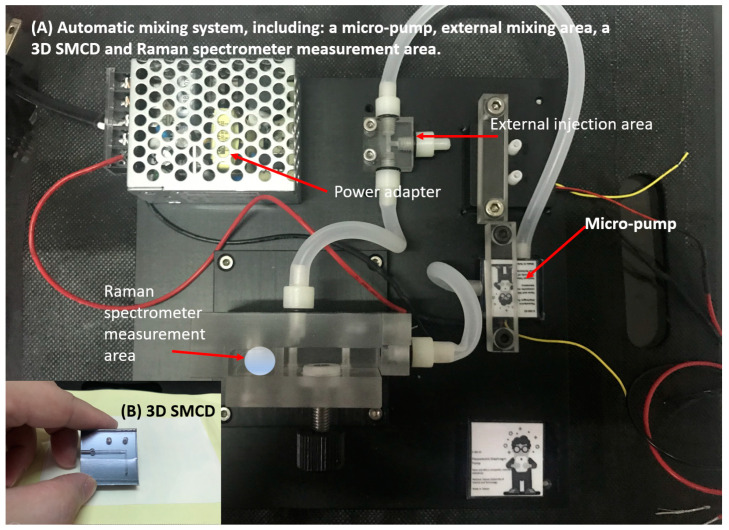
The automatic mixing system included a micropump, an external mixing area, a 3D SMCD, and a Raman spectrometer measurement area.

Blood was introduced into an automatic circulation system featuring an L-shaped microchannel region with a characteristic through-hole channel with a width of 750–1000 μm and a depth of 100 μm. As blood enters the internal microfluidic channel of the 3D SMCD device, the flow and rate can be precisely controlled by the micropump. This controlled flow reaches the mixing area, ensuring effective mixing for optimal performance. This setup is designed to facilitate convenient irradiation sampling by the laser light element of the Raman spectrometer, enhancing the overall efficiency of the system. The Raman spectroscopy system was integrated into the lower mixing zone of the 3D SMCD. The characteristic spectra were obtained from the Raman spectroscopy signals for blood clotting measurement, as shown in Figure 2. As evidenced by the Raman spectroscopy signals obtained in this study’s experimental results, successful acquisition of biological component information was collected.

**Figure 3:**
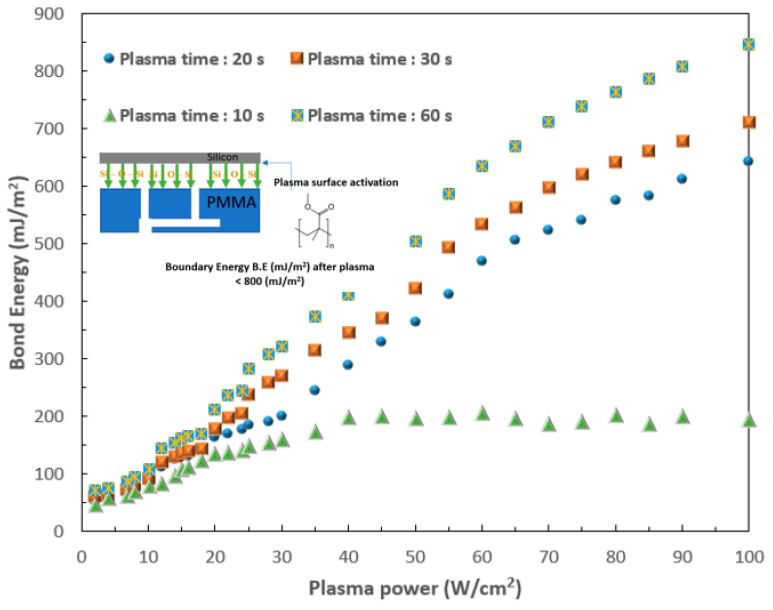
Curve showing the trend of the plasma power and bonding energy for the 3D SMCD.

The results demonstrate the successful application of this novel 3D SMCD, integrated with a Raman spectroscopy system, for blood clotting detection. The low-temperature bonding process utilized for the fabrication of the 3D SMCD, which featured a sandwich structure composed of PMMA/silicon/PMMA, achieved the bonding of heterogeneous materials while eliminating internal stresses. This also helps prevent product breakage or blood leakage. The 3D SMCD bonding was performed with different plasma times and plasma powers to achieve a high bonding energy. When the plasma treatment time was only 10 s, even at a high plasma power, the bonding energy reached only ~200 mJ/m^2^. The highest bonding strength was achieved after 60 s of plasma treatment time at a plasma power of 100 W/cm^2^. These bonding parameters provide a maximum bonding surface energy value of 841 mJ/m^2^, ensuring no leakage during blood measurements, as shown in Figure 3.

### 2.2. Blood Sample Preparation

All blood samples used in this study were obtained with the approval of the institutional review board, from blood donors at the National Cheng Kung University Hospital in Taiwan. Three types of samples were extracted from each blood specimen. None of the normal samples used in this study were subjected to any chemical or biological treatment. Blood samples were drawn using a syringe and then dropped in the upper hole of the 3D SMCD (100 μL). All the blood sample experiments were conducted at room temperature (RT). The whole blood obtained from healthy individuals typically had a PT of 10.0–12.5 s. In contrast, in abnormal blood, which had been obtained from patients receiving anticoagulant drugs, the coagulation factor required to bind to calcium (Ca^++^) ions in the blood, called factor IV was deficient, so the clotting time was longer than 12.5 s. Factor IV, also known as calcium (Ca^++^), plays a crucial role in coagulation, transforming liquid blood into the non-flowing gel essential for hemostasis. Abnormal blood samples in this study represented cases of Factor IV dysfunction often seen in patients on anticoagulant drugs, which compromise the blood’s clotting ability. In the experimental protocol, a calcium-containing coagulant was mixed with the blood at a ratio of 2.8:1. To meet the requirements of clinical practice, the prepared whole blood and plasma samples were also examined, as shown in Figure 1C.

## 3. Raman Spectroscopy

**Figure 4:**
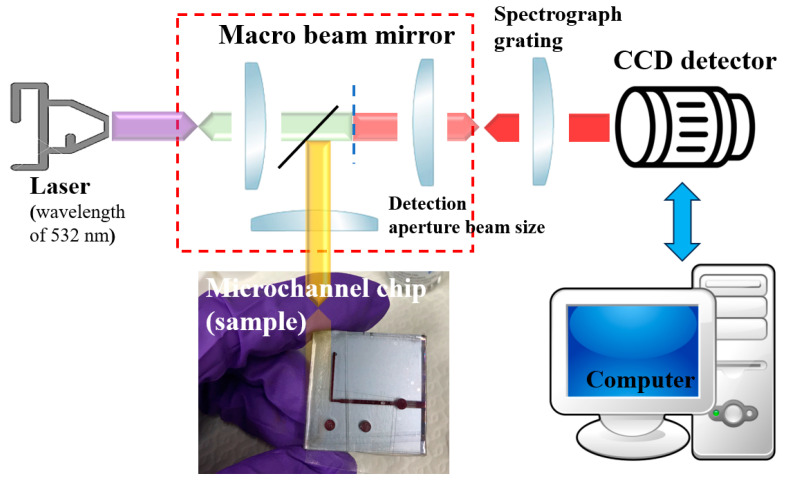
Micro-Raman spectroscopic system and 3D SMCD (sample).

Raman spectroscopy was performed at room temperature (RT) using a custom-made micro-Raman spectroscopy system consisting of a spectrometer (Princeton Instruments Acton Spectra Pro 2500i, Princeton Instruments, Trenton, NJ, USA), a liquid-nitrogen-cooled CCD detector (Princeton Instruments Acton 7508-0002, Princeton Instruments, Trenton, NJ, USA), four objective lenses from 20× to 100×, and a solid-state green laser (wavelength of 532 nm) as its excitation light source with the laser output power of 2 mW. To avoid laser heating damage to the blood samples, each Raman spectrum was recorded five times with an integration of 3 s. The spectral range was set to be between 400 and 2000 cm^−1^, as shown in Figure 3. The positions of the Raman peaks were characterized and calibrated with the measurements of the Si(100) substrate under the same conditions, and the background intensity was subtracted. In addition, the Raman spectra were normalized to the characteristic peak for whole blood at 1562 cm^−1^ [15], as shown in Figure 4.

## 4. Results and Discussion

### 4.1. Raman Spectrum of Whole Blood

**Figure 5:**
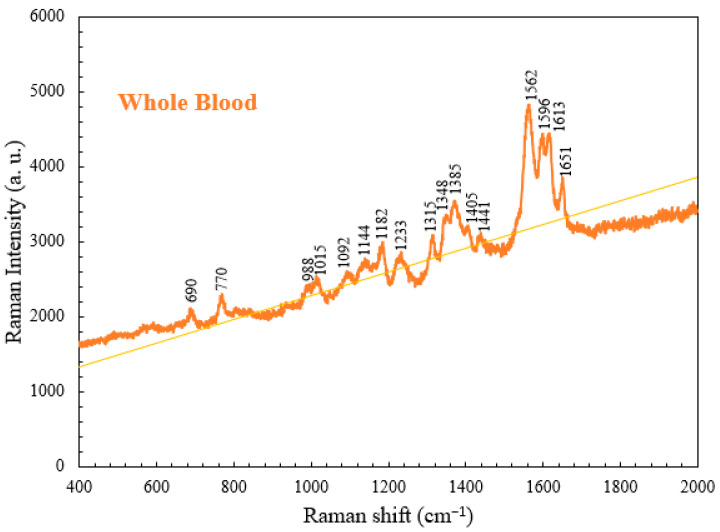
Raman spectrum for whole blood obtained at RT.

Based on the literature review and matching inquiries in the Spectral Database for Organic Compounds [16], we found the most abundant component in the whole blood to be hemoglobin [17], followed by amino acid, lactic acid, lactate, and acetate. Hemoglobin exhibits characteristic peaks at 690, 989, 1015, 1182, 1233, 1315, and 1562–1649 cm^−1^. Hemoglobin constitutes 95% of the weight of a red blood cell and affects the oxygen-carrying capacity [18]. In clinical practice, the hemoglobin level can serve as a standard for the detection of anemia [19], and the blood oxygen level indicates the severity of COVID-19 disease [5]. Amino acid is a small molecule [20] that exhibits characteristic peaks at 770 and 1348 cm^−1^. Moreover, lactic acid and lactate exhibit characteristic peaks at 1092 and 1144 cm^−1^, respectively. Lactic acid is a metabolic product produced from carbohydrates, and blood lactic acid levels can serve as a standard for the diagnosis of lactic acidosis in clinical practice [21]. Lactate is a salt produced when lactic acid releases a hydron and reacts with a positively charged ion like sodium or potassium. An excessive lactate level is recognized as a potential cause of acidemia (acidosis) [22]. The main characteristic peak of acetate was located at 1441 cm^−1,^ as shown in Figure 5. Acetate exists in animal tissues, excreta, and blood in the form of free acid. Table 1 displays the characteristic Raman peaks and vibrational modes of the components found in whole blood [23]. These components may have lower concentrations in the blood or change post-coagulation, resulting in weaker and less distinguishable peaks. Furthermore, the spectral range between 600 cm^−^^1^ and 800 cm^−^^1^ may contain the characteristic peaks of other biological components such as hormones, antibodies, and enzymes. Some peaks revealed by the Raman detection results were weaker and more difficult to distinguish due to structural changes in the platelets during low-temperature storage and potential lipid loss.

### 4.2. Comparison of the Raman Spectra of Whole Blood and Abnormal Blood as a Function of Factor IV

**Figure 6:**
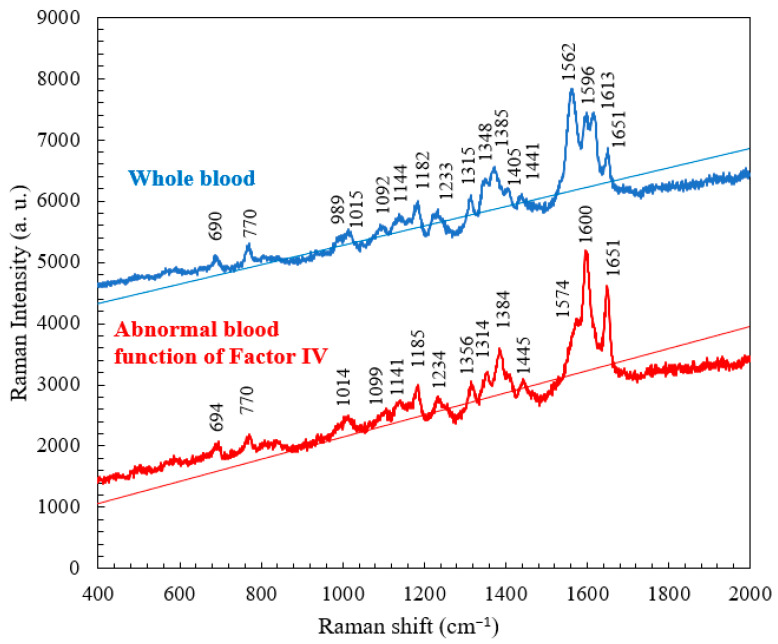
Raman spectra of whole blood and abnormal blood obtained at RT.

Comparison was made between the Raman spectra obtained from normal whole blood and blood with abnormal levels of Factor IV. The critical difference becomes apparent within the spectral range of 1562 to 1651 cm^−^^1^, as shown in Figure 6. The whole blood sample exhibited characteristic peaks at 1562 and 1613 cm^−1^, whereas the abnormal blood sample exhibited a characteristic peak at 1574 cm^−1^. This result suggests that the coagulation of hemoglobin occurred more easily in whole blood than in abnormal blood because of the shorter time period for clotting. Accordingly, the characteristic peaks for the aromatic ring breathing mode, C==N antisymmetric stretching, and pyrrole ring stretching were either absent or had shifted in position in the Raman spectrum of the abnormal blood sample [24]. Table 2 presents the characteristic Raman shifts and corresponding components for whole blood and abnormal Factor IV blood.

### 4.3. Comparison of the Raman Spectra of Abnormal (Factor IV) Blood with and without Coagulants

**Figure 7:**
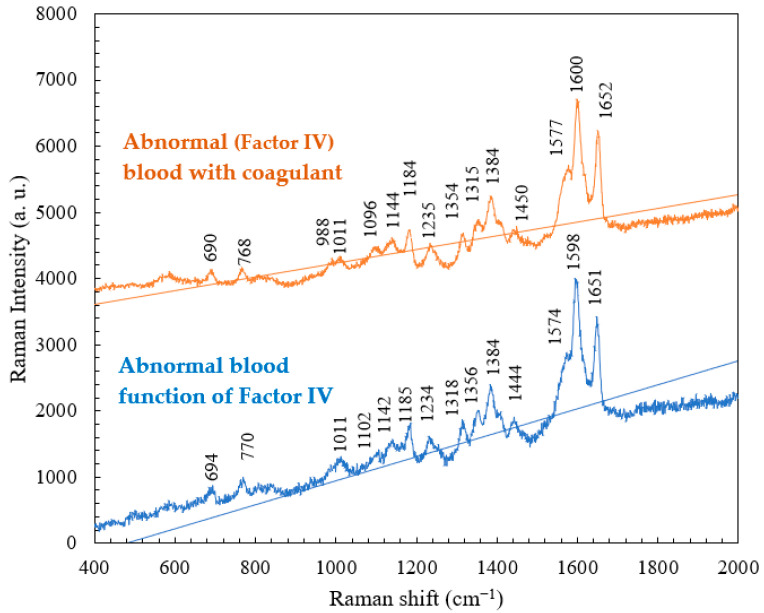
Raman spectra of whole blood without the coagulant and abnormal (Factor IV) blood with the coagulant obtained at RT.

A comparative study of abnormal (Factor IV) blood with and without a coagulant was conducted. The results, as shown in Figure 7, indicate that the intrinsic pathway can be initiated by events that take place within the lumen of the blood vessels. The intrinsic pathway requires only the elements (i.e., coagulation factors, Ca^++^, platelet surface, etc.) found within or intrinsic to the vascular system. In the comparison between abnormal blood samples with and without the coagulant, the abnormal (Factor IV) blood with coagulant had lower intensities at the characteristic peaks of 770, 1011, 1185, 1318, 1356, and 1384 cm^−1^ and higher intensities at 988 and 1577 cm^−1^. This change in intensity, without a shift in the peak position, is attributed to a (significant) change in the clotting time of hemoglobin. The addition of the coagulant shortens the clotting time, and therefore, the abnormal (Factor IV) blood, which originally took a long time to clot, became fully coagulated. Table 3 presents the major Raman shifts and corresponding components of normal blood without the coagulant and abnormal (Factor IV) blood with the coagulant. 

**Figure 8:**
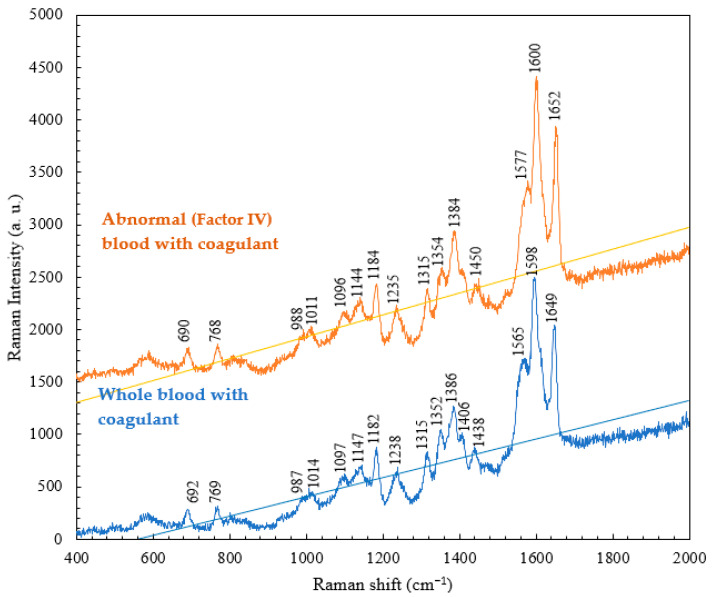
Raman spectra of normal blood with the coagulant and abnormal blood with the coagulant at RT.

In contrast, the results of a comparison of the Raman spectra of whole blood with the coagulant and abnormal (Factor IV) blood with the coagulant are presented in Figure 8. The characteristic peaks of the abnormal blood with the coagulant are similar to those of whole blood with the coagulant. It can be seen that the addition of the coagulant to the abnormal blood resulted in the wavenumber and intensities of its characteristic peaks gradually approaching those of the characteristic peaks of the whole blood with the coagulant. This indicates that the coagulation cascades were commonly shared in individual whole blood and abnormal (Factor IV) blood samples, but the time required for complete coagulation varied. In addition, the presence of the coagulant effectively shortened the time period for the clotting of abnormal blood, leading to full coagulation. 

In the experiments, blood samples were placed in the 3D SMCD and treated for approximately 15 s. Additionally, we measured the prothrombin time (PT), a method for assessing blood clotting ability, by adding Ca^++^ to the microfluidic device. To achieve rapid clotting, blood was automatically circulated within the mixing area. This process was completed in about 3 s. Some components may have lower concentrations in the blood or change post-coagulation, resulting in weaker and less distinguishable peaks. Furthermore, the spectral range between 800 cm^−^^1^ and 900 cm^−^^1^ may encompass characteristic peaks of other biological components such as hormones, antibodies, and enzymes. The Raman detection results revealed that, due to structural changes in the platelets during low-temperature storage and potential lipid loss, some peaks were weaker and less distinguishable.

### 4.4. Comparison of the Raman Spectra of Whole Blood, Plasma, and Serum

**Figure 9:**
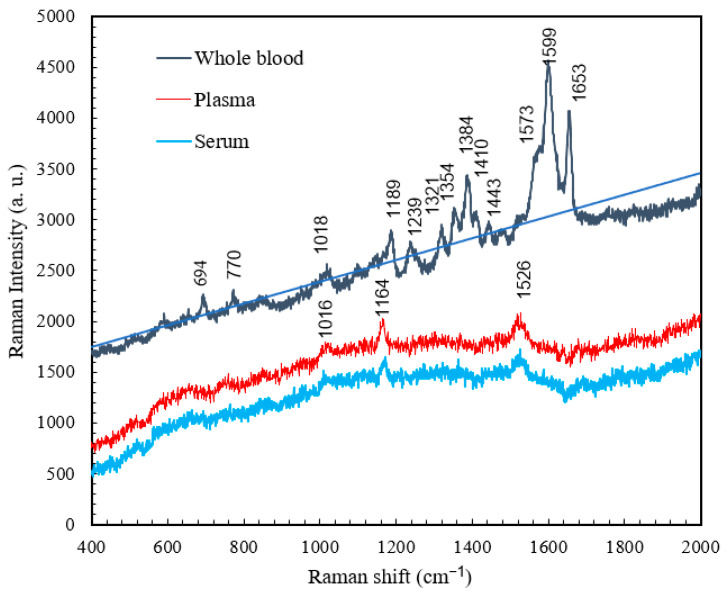
Raman spectra for whole blood, plasma, and serum obtained at RT.

Whole blood refers to normal blood that contains anticoagulants to inhibit coagulation. The characteristic peaks of whole blood have lower intensities than those of non-anticoagulated whole blood because of the absence of coagulation in whole blood, which prevents its molecules from achieving resonance [25,26]. However, the overall spectra obtained for normal blood and whole blood were similar, as shown in Figure 9. In contrast, the plasma and serum exhibited characteristic peaks only at 1016, 1164, and 1526 cm^−1^ because only some small molecules remained after the removal of blood cells. It has been suggested that the characteristic peaks of plasma and serum correspond to β-carotene [27], which only exhibits molecular resonance at low frequencies. In clinical applications, changes in the characteristic β-carotene peaks in the Raman spectra of serum and plasma can be used to diagnose certain diseases, such as thyroid disease and chronic renal failure. It has been recommended that the Raman shift of β-carotene can be used for diagnosing chronic renal failure and thyroid disease [28]. Table 4, Table 5 and Table 6 present the major Raman shifts and corresponding components for whole blood [22], plasma [28], and serum [29].

## 5. Contributions and Achievements

This study utilizes Raman spectroscopy for a detailed biological analysis of various blood samples (whole blood, plasma, and serum). The examination of blood components like hemoglobin, amino acids, lactic acid, lactate, acetate, and β-carotene offers insights into disease diagnosis and screening. The integration of the 3D SMCD enhances disease and biomarker detection by facilitating controlled mixing of blood and calcium coagulants, ensuring secure measurements without leakage. This approach combining Raman spectroscopy with the 3D SMCD is cost-effective and easy to operate, making it a practical alternative for application in medical settings. Additionally, valuable insights into coagulation pathways can be gained by comparing Raman spectra of abnormal blood with and without a coagulant, providing information about hemoglobin clotting times and contributing to a deeper understanding of blood coagulation dynamics. The research results point out the significance of β-carotene in medical testing, particularly in serum and plasma samples, because its molecular resonance at low frequencies can act as an indicator for diagnosing specific diseases such as thyroid disease and chronic renal failure.

## 6. Conclusions

We conducted a study utilizing a Raman spectrometer (excitation wavelength—532 nm) to analyze blood components in a 3D SMCD with both upper and lower mixing zones [30]. The combination of Raman spectroscopy with the microfluidic device allowed for more precise analysis and retrieval of information about the blood components. This method proved to be effective for measuring Raman spectral signals of whole human blood, offering a cost-effective and simple-to-operate alternative to high-precision confocal micro-Raman systems. Additionally, short-wavelength Raman scattering spectroscopy was employed to detect samples of whole blood, abnormal blood, plasma, and serum. Analysis of the biological characteristics detected in each sample type yielded several key conclusions and new findings. Firstly, the addition of a coagulant gradually changed the intensities and peak position of the characteristic peaks of abnormal blood, resembling those of normal blood with a coagulant. This behavior suggests that blood clotting affects the locations and intensities of the characteristic peaks associated with hemoglobin. Secondly, the results for abnormal blood without a coagulant differed from those of whole blood without a coagulant, creating strong differences at low-frequency vibration modes, which can serve as potential diagnostic fingerprints. Lastly, hemoglobin is not the main molecule in serum and plasma that resonates in Raman spectroscopy, but β-carotene exhibits molecular resonance at low frequencies, indicating its potential importance in medical testing.

In conclusion, our study highlights the significant contributions of integrating the 3D SMCD with Raman spectroscopy for blood sample analysis. Through our experimentation, we demonstrated the efficacy of this innovative approach in providing secure measurements without leakage and ensuring reliable and accurate results. The 3D SMCD, overcoming challenges in standard liquid sample systems, emerges as a robust platform for precise blood analysis. This study reinforces the practical advantages of our method and underscores its potential impact on advancing blood testing methodologies.

## Figures and Tables

**Table 1:** Characteristic peaks and corresponding vibrational modes of the components of whole blood.

Raman Shift (cm^−1^)	Vibrational Mode	Component
690	C-C-N bending	Hemoglobin
770	Ring vibrations	Tryptophan
989	Aromatic ring breathing	Hemoglobin
1015	Aromatic ring breathing	Hemoglobin
1092	C-O vibrations	Lactic acid
1144	CH_3_ rocking, C-O vibrations	Lactate
1182	C-C stretching	Hemoglobin
1233	Ferrous low spin	Hemoglobin
1315	C-C stretching	Hemoglobin
1348	C-H bending	Tryptophan
1385	CH_3_ symmetric stretching	Heme
1405	C==N antisymmetric stretching	Heme
1441	CH_2_ bending	Acetates
1562	Pyrrole ring stretching vibrations	Hemoglobin
1596	C==N antisymmetric stretching and	Hemoglobin
1613	C-H bending	Hemoglobin
1651	Ferrous low spin	Hemoglobin

**Table 2:** Major Raman shifts and corresponding components for whole blood and abnormal Factor IV blood.

Normal Raman Shift (cm^−1^)	Abnormal Blood Raman Shift (cm^−1^)	Vibrational Blood Mode	Component
989	No	Aromatic ring breathing	Hemoglobin
1315	1314	C-C stretching	Hemoglobin
1348	1356	C-H bending	Tryptophan
1562	1574	Pyrrole ring stretching vibrations	Hemoglobin
1596	1600	C==N antisymmetric stretching	Hemoglobin
1613	No	C-H bending	Hemoglobin
1651	1651	Ferrous low spin	Hemoglobin

**Table 3:** Major Raman shifts and corresponding components for abnormal blood samples with and without coagulants.

Abnormal Blood Raman Shift (cm^−1^)	Abnormal Blood with Coagulant Raman Shift (cm^−1^)	Vibrational Mode	Component
770	768	Ring vibrations	Tryptophan
1185	1184	C-C stretching	Hemoglobin
1318	1315	C-C stretching	Hemoglobin
1356	1354	C-H bending	Tryptophan
1574	1577	Pyrrole ring stretching vibrations	Hemoglobin

**Table 4:** Major Raman shifts and corresponding components for whole blood.

Raman Shift (cm^−1^)	Vibrational Blood Mode	Component
694	C-C-N bending	Hemoglobin
770	Ring vibrations	Tryptophan
1018	Aromatic ring breathing	Hemoglobin
1189	C-C stretching	Hemoglobin
1239	Ferrous low spin	Hemoglobin
1321	C-C stretching	Hemoglobin
1354	C-H bending	Tryptophan
1384	CH_3_ symmetric stretch	Heme
1410	C==N antisymmetric streching	Heme
1443	CH_2_ bending	Acetates
1599	C-H bending	Hemoglobin
1653	Ferrous low spin	Hemoglobin

**Table 5:** Major Raman shifts and corresponding components for plasma.

Raman Shift (cm^−1^)	Vibrational Blood Mode	Component
1016	C-H bending	β-Carotene
1164	C-C stretching	β-Carotene
1526	C-C stretching	β-Carotene

**Table 6:** Major Raman shifts and corresponding components for serum [29].

Raman Shift (cm^−1^)	Vibrational Blood Mode	Component
1013	C-H bending	β-Carotene
1172	C-C stretching	β-Carotene
1526	C-C stretching	β-Carotene

## Data Availability

Data are contained within the article.
